# Concepts of Double Hit and Triple Hit Disease in Multiple Myeloma, Entity and Prognostic Significance

**DOI:** 10.1038/s41598-020-62885-0

**Published:** 2020-04-06

**Authors:** Mehmet Baysal, Ufuk Demirci, Elif Umit, Hakki Onur Kirkizlar, Emine Ikbal Atli, Hakan Gurkan, Sedanur Karaman Gulsaran, Volkan Bas, Cisem Mail, Ahmet Muzaffer Demir

**Affiliations:** 1 Trakya University Faculty of Medicine, Department of Hematology, Edirne, Turkey; 2 Trakya University Faculty of Medicine, Department of Medical Genetics, Edirne, Turkey

**Keywords:** Myeloma, Translational research

## Abstract

Risk assessment in newly diagnosed multiple myeloma patients (NDMM) is the first and the most crucial determinant of treatment. With the utilization of FISH analysis as a part of routine practice, high risk Multiple Myeloma (MM) is defined as having at least one of the mutations related with poor prognosis including; t(4;14) t(14;16), t(14;20), del 17p, p53 mutation, gain 1q and del 1p. M-Smart MM risk stratification guideline by Mayo Clinic has proposed a concept similar to high grade lymphomas. Having two of the high risk genetic abnormalities were defined as double hit MM and having any three as triple hit MM. Based on these definitions which may bring a much more clinically relatable understanding in MM prognosis, we aimed to assess our database regarding these two concepts and their probable significance in terms of outcome and prognosis. We retrospectively evaluated 159 newly diagnosed multiple myeloma patients and their clinical course. Among these patients; twenty-four patients have one high risk determinant and also seven and two patients were classified as double hit MM and triple hit MM respectively. Overall survival (OS) of the patients with double hit MM was 6 months, 32.0 months for patients with single high risk abnormality and 57.0 months for patients with no high risk abnormality. Univariate analysis showed that Double Hit and Triple Hit MM is a predictive of low OS. Hazard Ratio of patients with one high risk abnormality was 1.42, double-hit MM patients was 5.55, and triple-hit MM patients was 7.3. Despite the development of novel drugs and their effects of prolonging survival, the treatment has not been individualized. Understanding the biology of each patient as a unique process will be the success of the treatment. As it is known that some MM patients harbor high risk genetic abnormalities according to FISH analysis, we can continue the argument that some patients bring an even higher risk and that can be defined as double or triple hit MM.

## Introduction

Risk stratification in hematological malignancies has been of particular prognostic importance with the availability of novel and sophisticated treatments. These risk indicators are incorporated in the nomenclature and treatment of certain lymphoma and leukemia types. Multiple myeloma (MM) accounts for 10% of all hematological cancers and in addition to the biochemical biomarkers, cytogenetic and molecular indicators have been accepted as crucial components of assessment^1^. Risk stratification of MM is generally based on Floresence *in Situ* Hybridization (FISH) method^2^. High risk MM is defined as having at least one of the mutations related with poor prognosis including; t(4;14) t(14;16), t(14;20), del 17p, p53 mutation, gain 1q and del 1p. M-Smart MM risk stratification guidelines from Mayo Clinic proposed a fresh point of view as having two of the high risk genetic abnormalities to be named as double hit MM and having any three as triple hit MM^3^. From this perspective, double or triple hit MM might be related with even poorer outcomes^4^. As more risk stratification tools are developed with sophisticated instruments including thorough genetic assessment, the initial and consecutive management of poor risk group patients are not distinctly established. Although life expectancy of patients with MM has increased with new treatment modalities, MM is still accepted as incurable and eventually all patients relapse. Therefore, it is important to notice which patient may bear a higher risk in the initial diagnostic period. In our study, we aimed to evaluate the frequency and the significance of high-risk abnormalities, double-hit and triple-hit in newly diagnosed MM patients.

## Patients and Methods

Data of 159 patients diagnosed with MM between May 2013 and December 2018 in Trakya University Medical Faculty evaluated in a retrospective manner. We included all MM patients diagnosed within the date range mentioned above at our center. Only patients diagnosed with Plasma Cell Leukemia were excluded from the study. Plasma cell leukemia was defined at least having more than 20% clonal plasma cell in the peripheral blood^5^. First line treatment agents, International Staging System (ISS), demographic and clinical characteristics and survival periods were recorded from files. Bone marrow biopsy was performed from each patient at diagnosis and a single sample was taken for FISH examination. Interphase FISH is used as a molecular cytogenetic tool for the identification of recurrent genetic abnormalities with major prognostic impact and predictive outcome in MM. MM has also been successfully studied by interphase FISH, because this is an assay that can be done in nondividing cells. On slides which had been prepared from cultured bone marrow cells, the FISH studies was carried out using standardized protocols, according to the manufacturer’s instructions (Cytocell, Cambridge, UK). Bone marrow biopsies performed and analyzed at diagnosis.FISH analyses for TP53/CEN17, D13S319 for 13q14, 13q34 (LSI13q34), t(4;14) (p16;q32) (LSI FGFR3/IGH Dual Color, Dual Fusion Translocation Probe Set), t(11;14) (q13;q32) (LSI CCND1/ IGH Dual Color, Dual Fusion Translocation Probe Set), t(14;16)(q32;q23), t(14;20)(q32;q12) (LSI IGH/ MAF Dual Color, Dual Fusion Translocation Probe Set), t(14;20) (IGH/MAFB Dual Color, Dual Fusion), CKS1B/CDKN2C (P18) Amplification/Deletion Probe (CytoCell, Cambridge, UK), were performed on uncultured bone marrow samples. A total of two hundred nuclei were enumerated for each FISH panel probe and cut-off for detection of a deletion/ fusion signal in the normal control sample was taken as 3%. The evaluation of FISH signals was performed using a fluorescence microscope (Axio Imager. M1. Carl Zeiss, Germany) with the software Cytovision 3.6 (Leica Biosystems Nussloch GmbH 2020, Buffalo Grove, IL 60089 United States). At least 200 interphase nuclei were analyzed for each slide.

Double-hit MM is defined by the coexistence of two high-risk abnormalities; triple-hit MM was evaluated by coexistence of three high-risk abnormalities. The high-risk abnormality was evaluated according to the latest m-smart guidelines (3). Lenalidomide treatment in the first line setting has not been reimbursed in Turkey’s health care system; therefore, bortezomib-based combinations were used as first line. Autologous hematopoietic stem cell transplantation (AHSCT) was performed in patients who were in remission with first-line treatment and fit to tolerate.

The study was conducted in accordance with the Declaration of Helsinki Ethical Principles for Medical Research. All Patients gave and signed informed consent. This study was approved by Trakya University ethical committee (Ethical Approval Number 2019–57).

Statistical analyses were performed using SPSS PC Ver.22 (IBM © SPSS Inc. USA). Descriptive statistics were given as number, percentage and arithmetic mean ± standard deviation (minimum -maximum). A two sided p value less than 0.05 considered significant. Overall survival (OS) was defined as time from diagnosis of MM to death. To evaluate overall survival; Kaplan-Meier overall survival (OS) estimates were calculated. Log rank test and Cox regression analysis were performed to evaluate estimate hazard ratios (HR) and 95% Confidence Intervals (CI). We also adjusted cox regression analysis for AHSCT and ISS.

## Results

### General features of patients

Mean age at diagnosis was 64.62 ± 11.07 years. Seventy six (47.7%) patients were male and 83 (52.2%) were female. 61.6% of the patients have received a combination regimen with bortezomib, cyclophosphamide, dexamethasone (CyBorD) in the first line setting because of the national regularities. Seventeen percent of the patients received bortezomib dexamethasone and 1.9% of the patients received bortezomib, thalidomide and dexamethasone. 47 of the 159 patients (29.6%) proceeded with AHSCT in the upfront setting. (Detailed information of the treatment modalities and clinical characteristics were summarized in Table 1).

**Table 1:** Demographic Clinical Characteristics of the Patients.

	No high risk Cytogenetic abnormality (n = 126)	One high Risk Cytogenetic Abnormality (n = 24)	Two High Risk Cytogenetic Abnormalities (n = 7)	Three High Risk Cytogenetic Abnormalities (n = 2)	Total (n = 159)
**Demographic and Clinical Characteristics of the Patients**					
Age (years)	64.5 ± 11.5	65.4 ± 9.4	61.6 ± 9.9	71.0 ± 4.2	64.6 ± 11.1
Gender (male/female)	60/66	12/12	2/5	2/0	76/83
ISS stage at diagnosis					
Stage 1	70 (%58.8)	1 (%4.3)	0	0	71(%47.0)
Stage 2	44 (%37.0)	9 (%34.8)	1 (%14.3)	0	53(%35.1)
Stage 3	5 (%4.2)	14 (%60.9)	6 (%85.7)	2 (%100.0)	27(%17.9
First Line Treatment					
VCD	75 (%59.5)	14(%58.3)	7 (%100.0)	2(%100.0)	98(%61.6)
VD	25 (%19.8)	3 (%12.5)	0	0	28(%17.6)
VTD-PACE	0	3 (%12.5)	0	0	3(%1.9)
Others	26 (%20.7)	4 (%16.7)	0	0	30(%18.9)
Upfront ASCT	39 (%31.0)	7 (%29.2)	1 (%14.3)	0	47(%29.6)
Follow Up Time (moths)	39.3 (5–69)	26.8 (4–54)	6.6 (1–10)	—	
Overall Survival (months)	57.0 ± 9.6	32.0 ± 25.6	6 ± 4.2	—	50.0 ± 11.0

### Genetic risk profile and concepts

**Figure 1:**
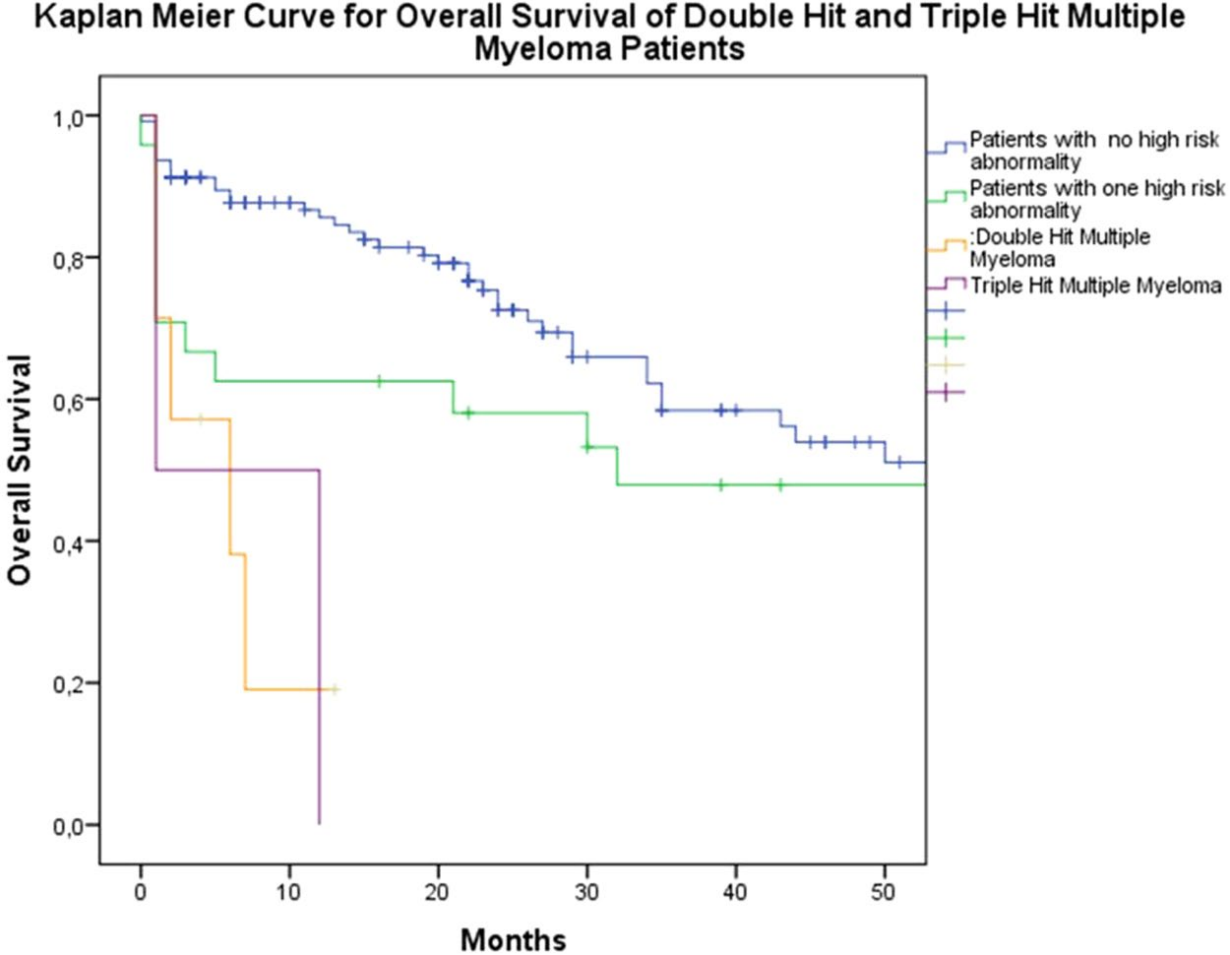
Kaplan Meier Overall Survival Analysis of the Patients with High Risk Abnormalities; including double hit and triple hit patients.

Twenty-four patients were observed to have one high risk, 7 patients with two high risk and 2 patients with three high risk determiners. Overall survival of the patients with two high risk abnormalities was 6 ± 4.2 months, while 32.0 ± 25.6 months for patients with single high risk abnormality and 57.0 ± 9.6 months for patients with no high risk abnormality. The adverse effects of high risk abnormalities on overall survival were assessed with cox regression analysis. HR of patients with one high risk abnormality was 1.42 (95% CI 0.77–2.63) (p = 0.255), while HR of double-hit MM patients was 5.55 (95% CI 2.09–14.58) (p = 0.001) and finally, HR of triple-hit MM patients was 7.34 (% 95 CI 1.72–31.14) (p = 0.007). OS estimates and HR values of high risk abnormalities were summarized in Table 2. Kaplan-Meier OS Estimates were depicted in Fig. 1. In double hit myeloma patients four patients has the co-occurrence of 17p deletion and t (4;14), one patient has 17p deletion and gain of 1q, one patient has 17p deletion and t (14;16), one patient has gain of 1q and t(14;16). In triple hit MM patients one patient has 17p deletion, t(4;14) and t (14;16), one patient has 17 p deletion, t (4;14) and t (14;20).

**Table 2:** Cox Regression Models for the Effect of High Risk Abnormalities on Overall Survival and Hazard Ratios.

	Hazard Ratio (95% CI)	p Value
Patients With One High Risk Cytogenetic Abnormality	1.42 (% 95 CI 0.77–2.63)	0.255
Patients With Two High Risk Cytogenetic Abnormalities	5.55 (% 95 CI 2.09–14.58)	0.001
Patients With Three High Risk Cytogenetic Abnormalities	7.34 (% 95 CI 1.72–31.14)	0.007

Twenty-one patients with 17p deletion, ten patients with t (4;14), nine patients with t (14;16), four patients with gain of 1q and one patient with t (14;20) were reported in our cohort. Eleven of the patients with 17p deletion (52.23%) had also p53 mutation. We evaluated the effects of each individual high risk abnormality on OS estimates and 17p deletion and gain of 1p were observed to have an effect on OS. The other high three high risk abnormality were not a factor for overall survival in cox regression analysis. HR and 95% CI values were summarized in Table 3.

**Table 3:** Cox regression models of each high risk abnormality on overall survival estimate and hazard ratios.

	Hazard Ratio (95% CI)	p Value
17p del (number:21)	0.47 (0,25–0,87)	0.017
Gain of 1q (number:4)	0.46 (0,44–0,63)	0.009
t (4;14) (number:10)	0.49 (0,20–1,11)	0.100
t (14;16) (number:9)	0.86 (0,34–2,18)	0.764
t (14;20) (number:1)	0.91 (0,85–9,73)	0.939

### Effect of treatments on double/triple Hit MM

Regarding the effect of ASCT on high-risk abnormalities, we stratified the data for ASCT patients with no high-risk abnormality seems to benefit more from ASCT. Hazard ratio for no high risk abnormality were 1.97 (95% CI 1.91–3.35, p value 0.012. n:39). Hazard ratio for one high risk abnormality were 0.20 (95% CI 0.47–0.85 p value 0.029 n:7). Hazard ratio for double hit myeloma was 0.30 (95% CI 0.65–1.37 p value 1.21 n:1). Hazard ratio for triple hit myeloma 0.98 (95% CI 0.19–5.13 p value 0,98 n:0). As the numbers of ASCT performed in patients with high risk abnormalities were low, these results are difficult to interpret. After adjusting for ISS HR for one high risk abnormality was 0.13 (95% CI 0.31–0.56 p value 0.006). HR for double hit myeloma was 0.19 (95% CI 0.43–0.90 p value 0.037). HR for triple hit myeloma was 0.75 (95% CI 0.14–3.86 p value 0.73). As the sample size of double and triple hit myeloma were small, the effect of ISS seems to be more in one high-risk abnormality group.

## Discussion

Since the first recognition of MM, the heterogeneity of the clinical course has been one of the major challenges, to predict the rapid progressing patients as well as gradual advancers. The prognosis has been suggested to be dependent on tumor burden (stage of the disease), patient’s condition and comorbidities, access to treatment and disease biology, which is the motive of our study, to predict how aggressive the disease is, for each unique patient^6^. To identify disease biology, certain perspectives have been proposed with substantial understanding of prognosis including bone marrow plasma cell immunophenotype and certain diversities, the rate and capacity of the plasma cell proliferation, the presence of plasma cells in circulation and cytogenetic abnormalities. At the time of diagnosis, it is recommended to determine specific cytogenetic abnormalities using FISH method. Due to the slow proliferation capacity of plasma cells in MM, FISH method rather than the metaphase cytogenetic method is regarded to be plausible for the detection of translocations in clonal cells^7^. One of the major disadvantages of FISH method is the dependency to the quantity of bone marrow plasma cell percentage. Interphase FISH method with plasma cell enrichment by CD 138 labelling rather than examining on cultured bone marrow samples are suggested for higher detection rates of genetic abnormalities^8,9^. To date, European Myeloma Network recommends Interphase FISH method for MM and states as acceptable, till plasma cell enrichment methods becomes more accessable and less costly^10^.

While the genetical abnormalities in these clonal cells contribute to the nature and aggressiveness of MM, tissue microenvironment, which is the interaction and response of the surrounding bone marrow to these malignant cells are thought to contribute to the poor prognosis of these patients^11–13^. In this perspective, some patients may harbor an ultra-high risk disease classified as double-hit or triple-hit MM. In a recent analysis of newly diagnosed MM patients, approximately one high risk abnormality was detected in 1 of every 4 patients while two high risk abnormalities were detected in 1 of every 33 patients^14^. Similar to these findings; we observed one high risk abnormality in 24 of the 159 patients and two high risk abnormalities were observed in 7 of the 159 patients. In the same study^12^, OS of patients with one high risk abnormality was 4.9 years while 3.0 years in patients with two high risk abnormalities. In our study, OS of patients with one high risk abnormality was 32 months and 6,0 months in patients with double-hit MM patients.

Besides FISH method, Next generation sequencing (NGS) method has been more and more popular in MM as well as in all other hematological malignancies and premalignant conditions. Pointed to be the, future of cancer research and especially in MM, the complexity of the method with being expensive than FISH, NGS needs time to become the next standard of care for MM. Studies using NGS on MM have demonstrated quite intriguing results. In a study categorizing patients using NGS have reported that OS is shorter in double-hit MM patients^15^. In an another recent analysis the authors put together the revised ISS, and Next Generation Sequencing based FISH analysis, the authors performed this study in 672 patients and high risk chromosomal abnormality is defined as deletion 17p, t(4;14), t(14;16). The authors also reported that patients with R-ISS-NGS stage 2 and 3 has higher risk of death compared to R-ISS-NGS stage 1^16^.

In another retrospective analysis, patients were defined as ultra-high risk MM having both a high risk abnormality detected with FISH analysis and ISS stage 3 disease. 120 of the 1461 patients (%8) had ultra-high risk MM^17^. Forty-one percent of the study population were reported to undergo upfront AHSCT, while in our data % 29,6 of our patients have underwent upfront AHSCT which is slightly less and may be explained by the fact that our cohort have reached remission harder with first line treatment due to the increased number of high risk patients. In a meta-analysis which includes data from NCRI Myeloma XI and MRC Myeloma IX trials; double-hit MM defined as co-occurrence of at least any two of t(4;14), t(14;16), t(14;20); gain(1q); del(17p) high risk abnormalities. The authors conclude that patients with double-hit MM particularly dismal prognosis comparing to standard risk patients^18^.

In a case report, double-hit MM was suggested in a rapidly progressing and poor prognosis patient as having both IGH / MYC and IGH / CCND1 translocations^19^. A similar case report demonstrated double-hit plasma cell leukemia patient with IGH/MYC and IGH/BCL2 translocations^20^. Anyhow, it is more appropriate to evaluate double-hit MM with the FISH method, as in our study, rather than with MYC and other associated translocations.

There are certain limitations of our study including the retrospective nature of our study, relatively small sample size and the use of FISH method without plasma cell enrichment, plasma cell labelling or CD 138 positive cells. In this context, we observed a limited number of double-hit and triple-hit MM patients. However, as a real life observation, similar to lymphoproliferative disorders, double or triple hit to certain genetical locations may alter the prognosis.

## Conclusion

Proteasome inhibitors and immunomodulatory drugs are the novel drugs that have changed the outcome and dramatically prolonged the survival of MM even in high risk patients. However, the standard approach to all patients regardless of their high risk potential remains an unsettled issue in the clinicians’ minds.

The evolution of monoclonal gammopathy of undetermined significance to overt MM with the addition of extra genetical evolution and instability step by step shows us a great example of cancer stem cell theory. Double or triple hit MM may find their place as the last ring in this theory. As double-hit or triple-hit lymphomas are accepted to need intensive treatment compared to standard risk patients, it may be attributed to MM as a concern that double hit or triple hit MM patients should also be treated more intensively. In our study, patients with one or two high-risk abnormalities had lower overall survival than patients with no high-risk abnormality.

Double Hit or Triple Hit MM should be better defined and described and this particular information shall lead the road to individualized therapy in MM.
